# Five-year competing-risk analysis of infectious and noninfectious complications after lung transplantation: Real-world evidence from a multicenter EHR-based cohort

**DOI:** 10.1016/j.jhlto.2026.100536

**Published:** 2026-03-10

**Authors:** German A. Contreras, George Golovko

**Affiliations:** a Department of Internal Medicine, Division of Transplant Infectious Diseases, Duke University, Durham, North Carolina; b Department of Pharmacology & Toxicology, University of Texas Medical Branch at Galveston, Galveston, Texas; c Center for Health and Clinical Outcomes Research, University of Texas Medical Branch at Galveston, Galveston, Texas

**Keywords:** competing risk, benchmark, lung transplantation, electronic health records, TriNetX

## Abstract

This study evaluates the 5-year incidence of major post-transplant outcomes using a competing-risks framework. We analyzed data from 10,648 lung transplantation (LTx) recipients using the TriNetX U.S. Collaborative Network (January 1, 2015, to December 31, 2024). The Aalen-Johansen method was used to estimate the cumulative incidence function (CIF) of post-transplant complications. Infectious complications had the highest 5-year CIF (30.88%) and steepest annual trend (5.79%), slightly exceeding noninfectious complications (30.28%, 5.69%). Mortality reached 10.62%, and malignancies 6.72%. Cytomegalovirus showed the highest infectious burden (CIF 17.02%, annual trend 3.40%), with a steep rise within the first 180 days. Bacterial pneumonia followed a slower but sustained trajectory (CIF 9.1%, 1.8% annually). Statistical analyses confirmed significant differences across all outcomes (*p* < 0.001). Infectious and noninfectious complications dominate the post-LTx landscape and follow distinct temporal trajectories. Our data underscore the need for dynamic, risk-based surveillance and individualized prevention strategies to optimize long–term transplant outcomes.

## Background

Lung transplantation (LTx) is a pivotal therapy for end–stage lung disease but is frequently complicated by infection, rejection, and comorbidities. Traditional survival models assume outcomes occur independently, overlooking that events such as death or graft failure preclude others.^1^ To address this, we used a competing-risks framework to characterize 5-year trajectories of infectious complications, noninfectious complications, malignancy, and mortality. This approach provides time–based clinical phenotypes while accounting for competing outcomes and focuses on temporal patterns and cumulative syndrome-level burden consistent with electronic health record (EHR)-derived data.

## Methods

### Study cohort and data source

We identified a cohort of individuals ≥18 years old with only LTx history from January 1, 2015, to December 31, 2024, excluding those with multiple organs, retransplanted or bone/stem cell transplants from the TriNetX U.S. database. The study period encompasses the interval between the LTx index date and the end of 1,825 days of follow-up (5 years), with censoring at last encounter available in TriNext. The platform aggregates deidentified EHRs from 86 million patients across 104 academic and community health systems and standardizes data by using recognized health care coding systems. A detailed description of the diagnosis, procedure, medication, and outcome International Classification of Diseases (ICD)-10 and current procedural terminology codes used in this study is provided in the Supplementary Material (Supplement Tables 1 and 2). We only included ICD-10 codes representing active infection, and codes denoting colonization or carrier status were excluded (Supplemental Tables 1 and 2). While these codes identify active infectious syndromes, they cannot distinguish invasive from noninvasive disease or provide organism-level or resistance information; therefore, outcomes are interpreted at the syndrome level. Although the study period aligns with the ICD-10 era, legacy ICD-9-CM data from some institutions may be present. TriNetX converts ICD-9-CM diagnoses to ICD-10-CM using general equivalence mappings-based mappings supplemented by custom algorithms and manual curation (Supplemental Material Methods). This standardized approach improves temporal comparability across the dataset. This study was approved by the University of Texas Medical Branch Institutional Review Board (20-0085).

### Statistical analysis

Baseline characteristics were summarized as means ± SDs (standard deviations) for continuous variables and frequencies (%) for categorical variables. Competing risks were analyzed using the Aalen-Johansen method within TriNetX, which estimates cumulative incidence functions (CIF) for infection complications, noninfectious complications, malignancy, and death. This approach enables us to estimate absolute risk over time for each mutually exclusive outcome, rather than evaluating covariate effects, which is the primary utility of the Fine-Gray model. Each study outcome was constructed using a distinct combination of ICD-10 codes, as detailed in Supplementary Tables 1 and 2. Infectious complications, noninfectious complications, malignancy, and death were therefore defined using separate, nonoverlapping code groupings to ensure clear delineation among mutually exclusive outcome categories. These standardized definitions allowed consistent classification of events across participating institutions and formed the basis for configuring competing risks within the Aalen-Johansen framework. The Aalen-Johansen estimator calculates the CIF of each mutually exclusive outcome. Thus, if a patient first developed a noninfectious complication, they contributed to that outcome’s incidence but were no longer considered at risk for an infectious event, and vice versa. This approach avoids the overestimation that occurs when competing events are handled as simple censoring and provides more clinically realistic estimates of event probability over time. Patients without an event were censored at their last clinical encounter. CIF estimates with 95% confidence intervals were generated using the R Survival package (v3.2-3), with Kaplan-Meier used for survival analyses (Supplemental Material). Group comparisons employed analysis of variance, Kruskal-Wallis, and Bonferroni-adjusted Mann-Whitney U tests (*p* < 0.05). Temporal progression was assessed using trend analysis, with total increase (absolute change) and annual trend (year-over-year percentage change) as key metrics.^2^, ^3^ The former represents the absolute change in a variable over a specified period, indicating how much it has increased or decreased. This is useful for understanding long-term shifts, such as the overall rise in outcomes in LTx over a period. The annual trend over time reflects the year-over-year pattern of change, often expressed as a percentage. These temporal trend metrics help clinicians understand not only the overall burden of complications but also the pace at which they accumulate over time. This information supports more informed surveillance planning, allowing providers to anticipate periods of heightened risk, adjust follow-up intensity, and tailor preventive strategies to the evolving clinical trajectory of lung transplant recipients. Analyses were conducted in Python with NumPy and Matplotlib.

#### Missing data

TriNetX excludes patients from specific analytic steps when required values—such as event status or time-to-event—are not available. Patients without documented events or corresponding time were therefore not included in the competing-risk calculations. The platform does not impute missing data, and analyses rely solely on available-case information from contributing health systems.

## Results

**Figure 1:**
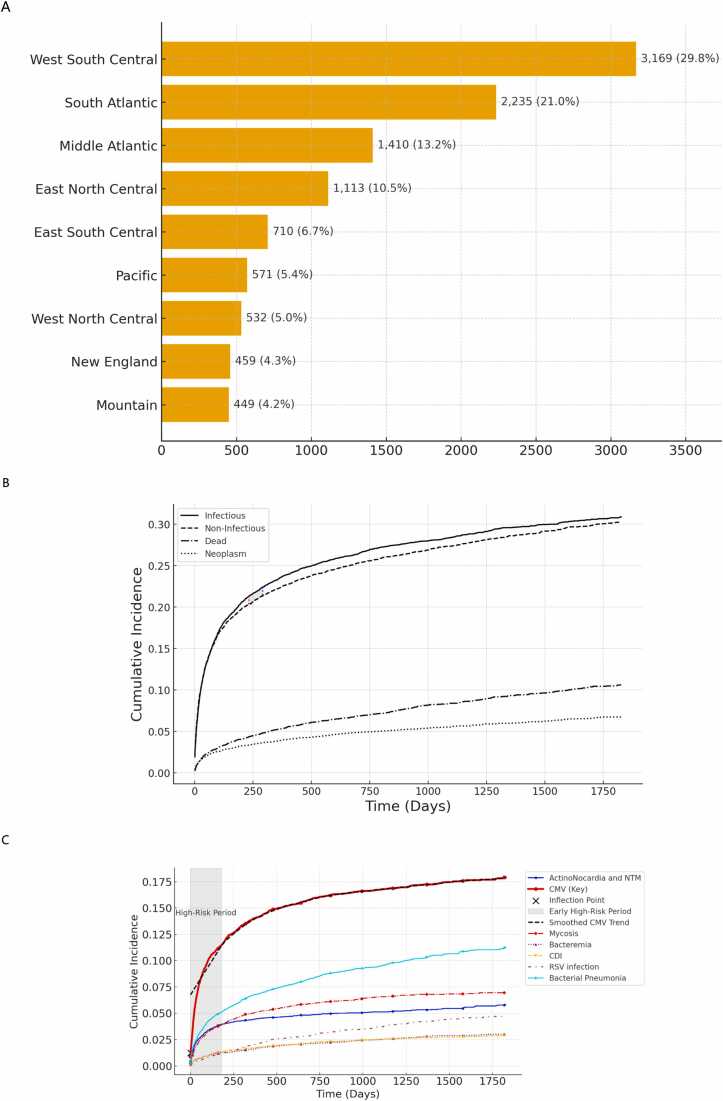
Geographic distribution of transplant recipients and cumulative incidence of major outcomes in lung transplant recipients. (A) Geographic distribution of LTx patients included in the analysis. (B) Five-year incidence of major complications and mortality in lung transplant recipients. (C) Five-year incidence of infection outcomes over time. (A) Shows the geographic distribution of lung transplant recipients in the cohort (*n* = 10,648). (B) This plot visualizes the cumulative incidence (gray lines) of a specified outcome over time, as estimated using the Aalen-Johansen method, demonstrating infectious and noninfectious events as the most prevalent outcomes across 5 years of follow-up. The divergence between infectious and noninfectious risks is first visually apparent at 232 days (red), but statistical significance is reached at 291 days (blue). The shaded region represents the transition phase. (C) The Aalen-Johansen cumulative incidence curve illustrates the distinct trajectory of cytomegalovirus (CMV) infection in solid organ transplant recipients. The CMV curve (red) is highlighted with a bold line, and the early high-risk period (0-180 days post-transplant) is shaded in gray to emphasize the critical window for infection surveillance and prophylaxis. A black X marker identifies the inflection point, indicating the steepest increase in cumulative incidence, which likely corresponds to the peak of viral replication. The dashed black line represents a LOWESS-smoothed trend, providing a clearer view of CMV progression. The visualization effectively captures the temporal dynamics of infection risks, facilitating a clear understanding of which conditions warrant the most attention—actinomycosis-*Nocardia* and NTM: actinomycosis, *Nocardia*, and nontuberculous mycobacteria. LTx, lung transplantation.

The study included 10,648 LTx, with a mean age of 58.7 years (SD ± 14). The majority were male (54%) and predominantly White (69%) (Supplemental Table 1). The cohort was geographically diverse, with the highest representation from the West South Central (29.7%) and South Atlantic (21.0%) regions, followed by the Middle Atlantic (13.2%) and East North Central (10.5%) regions (Figure 1A). Infectious disease burden before transplantation was notable, with 13% having a history of influenza or pneumonia, 3% fungal infections (aspergillosis or candidiasis), and 2% having a history of antimicrobial-resistant infections. Mental and behavioral disorders were also prevalent (24%), highlighting the psychosocial complexity of this population.

### Competing-risk analysis and trends of post-transplant outcomes

Infectious complications demonstrated the steepest trajectory, with a 5-year CIF of 30.9%, a total increase of 28.9%, and an annual trend of 5.8%. Noninfectious complications were similarly frequent (CIF 30.3%, increase 28.4%, annual trend 5.7%), but diverged from infections by day 291 (*p* = 0.0083), a separation that persisted through follow-up (Figure 1B, Tables 1 and 2), accumulated at a comparatively faster rate. Clinically, this transition aligns with the period when patients move beyond immediate postoperative risks and become increasingly susceptible to infection-related complications, underscoring the importance of targeted surveillance and prevention strategies during this interval. Mortality (CIF 10.6%, annual trend 2.1%) and malignancies (CIF 6.7%, annual trend 1.3%) rose more gradually, reflecting a steady but slower accumulation over time (Figure 1B, Tables 1 and 2). Interval changes differed significantly across outcomes [analysis of variance F = 14,080; Kruskal-Wallis H = 4,611; both *p* < 0.001], with all pairwise comparisons significant after Bonferroni correction (*p* < 0.001).

**Table 1:** Competing-Risk Analysis of Clinical Outcomes Within 5-Year Post-LTx Transplant

Study Variables	Patient count	% of cohort	Cumulative incidence	95% CI
*Outcome*				
Infectious^a^	2,592	24.34	30.88	23.62-38.42
Noninfectious^b^	2,513	23.60	30.28	22.86-38.06
Death	748	7.02	10.62	7.42-14.46
Neoplasm^c^	505	4.74	6.72	4.77-9.12

*Infectious outcome*				
Cytomegalovirus (CMV) disease^d^	1,461	13.72	17.91	12.18-24.54
Bacterial pneumonia^e^	805	7.56	11.26	6.96-16.69
Mycosis^f^	546	5.13	7.00	4.72-9.85
Actinomycosis, *Nocardia*, NTM^g^	468	4.39	5.83	3.70-8.62
RSV infection^h^	295	2.77	4.70	2.71-7.49
Bacteremia	209	1.96	3.01	1.82-4.65
*Clostridium difficile* infection^i^	208	1.95	2.88	1.81-4.32

Abbreviations: LTx, lung transplantation; NTM, nontuberculous mycobacteria; RSV, respiratory syncytial virus.

a CMV disease, bacterial pneumonia, bacteremia, mycosis, *C difficile* infection, RSV.

b Complications of transplanted organs and tissue (ICD-10 T86), Lung transplant rejection (ICD-10 T86.818), Heart-lung transplant rejection (ICD-10 T86.31), Bronchiolitis obliterans and bronchiolitis obliterans syndrome (ICD-10 J44.81), Pneumothorax and air leak (ICD-10 J93), Other pneumothorax and air leak (ICD-10 J93.8), Other pneumothorax (ICD-10 J93.83), Pneumothorax, unspecified (ICD-10 J93.9), Hemothorax (ICD-10 J94.2), and Pyothorax with fistula (ICD-10 J86.0)

c Melanoma and other malignant neoplasms of skin (ICD-10 C43-C44) and Malignant neoplasms of lymphoid, hematopoietic, and related tissue (ICD-10 C81-C96)

d Other cytomegaloviral diseases (ICD-10 B25.8), Cytomegaloviral disease (ICD-10 B25), Cytomegaloviral pneumonitis (ICD-10 B25.0), Cytomegaloviral disease (ICD-10 B25), Cytomegaloviral hepatitis (ICD-10 B25.1), Cytomegaloviral pancreatitis (ICD-10 B25.2), Cytomegaloviral mononucleosis with polyneuropathy (ICD-10 B27.11) and Cytomegaloviral mononucleosis with meningitis (ICD-10 B27.12)

e Bacterial pneumonia not elsewhere classified (ICD-10 J15), Unspecified bacterial pneumonia (ICD-10 J15.9), Lobar pneumonia, unspecified organism (ICD-10 J18.1), Bronchopneumonia, unspecified organism (ICD-10 J18.0) and Pneumonia in diseases classified elsewhere (ICD-10 J17).

f Coccidioidomycosis (ICD-10 B38), Histoplasmosis (ICD-10 B39), Blastomycosis (ICD-10 B40), Zygomycosis (ICD-10 B46), other mycoses, not elsewhere classified (ICD-10 B48), Aspergillosis (ICD-10 B44), Invasive pulmonary aspergillosis (ICD-10 B44.0), Unspecified Aspergillosis (ICD-10 B44), Other forms of Aspergillosis (ICD-10 B44.8), Other pulmonary Aspergillosis (ICD-10 B44.1) and disseminated aspergillosis (ICD-10 B44.7).

g Nocardiosis (ICD-10 A43), Nocardiosis, unspecified (ICD-10 A43.9), *Nocardia* sp. identified in Specimen by Organism-specific culture (Code 43365-6), *Nocardia* sp. identified in Isolate (Code 58596-2), Actinomycosis (ICD-10 A42), Other forms of nocardiosis (ICD-10 A43.8), Pulmonary nocardiosis (ICD-10 A43.0), Pulmonary actinomycosis (ICD-10 A42.0), and Pulmonary mycobacterial infection (ICD-10 A31.0).

h Acute bronchiolitis due to respiratory syncytial virus (ICD-10 J21.0), Acute bronchitis (ICD-10 J20), Acute bronchitis due to respiratory syncytial virus (ICD-10 J20.5), and Respiratory syncytial virus pneumonia (ICD-10 J12.1).

i Enterocolitis due to *C difficile* (ICD-10 A04.7), Enterocolitis due to *C difficile*, not specified as recurrent (ICD-10 A04.72), and Enterocolitis due to *C difficile*, recurrent (ICD-10 A04.71)

**Table 2:** Temporal Trend Analysis of Cumulative Incidence Outcomes in Lung Transplant Recipients

Study Variables	Total increase over time (%)	Annual trend rate (%)
Infectious^a^	28.95	5.79
Noninfectious^b^	28.43	5.69
Death	10.41	2.08
Neoplasm^c^	6.35	1.27
Infectious outcome		
Cytomegalovirus (CMV) disease^d^	17.02	3.40
Bacterial pneumonia^e^	10.96	2.19
Mycosis^f^	6.79	1.36
Actinomycosis, *Nocardia*, NTM^g^	5.39	1.08
RSV infection^h^	4.64	0.93
Bacteremia	2.87	0.57
*C difficile* infection^i^	2.82	0.56

Abbreviations: NTM, nontuberculous mycobacteria.

Interpretation of values: The table presents temporal trends in the cumulative incidence of various clinical outcomes among lung transplant recipients. The values reflect percentage increases over time and the annual trend rate. The total increase over time represents the absolute change in a variable over a specified period, showing how much it has grown or declined in total. The annual trend over time reflects the year-over-year pattern of change, often expressed as a percentage or rate. This helps in identifying consistent growth, seasonality, or fluctuations.

a CMV disease, bacterial pneumonia, bacteremia, mycosis, *C difficile* infection, RSV.

b Complications of transplanted organs and tissue (ICD-10 T86), Lung transplant rejection (ICD-10 T86.818), Heart-lung transplant rejection (ICD-10 T86.31), Bronchiolitis obliterans and bronchiolitis obliterans syndrome (ICD-10 J44.81), Pneumothorax and air leak (ICD-10 J93), Other pneumothorax and air leak (ICD-10 J93.8), Other pneumothorax (ICD-10 J93.83), Pneumothorax, unspecified (ICD-10 J93.9), Hemothorax (ICD-10 J94.2), and Pyothorax with fistula (ICD-10 J86.0)

c Melanoma and other malignant neoplasms of skin (ICD-10 C43-C44) and Malignant neoplasms of lymphoid, hematopoietic, and related tissue (ICD-10 C81-C96).

d Other cytomegaloviral diseases (ICD-10 B25.8), Cytomegaloviral disease (ICD-10 B25), Cytomegaloviral pneumonitis (ICD-10 B25.0), Cytomegaloviral disease (ICD-10 B25), Cytomegaloviral hepatitis (ICD-10 B25.1), Cytomegaloviral pancreatitis (ICD-10 B25.2), Cytomegaloviral mononucleosis with polyneuropathy (ICD-10 B27.11), and Cytomegaloviral mononucleosis with meningitis (ICD-10 B27.12).

e Bacterial pneumonia, not elsewhere classified (ICD-10 J15), Unspecified bacterial pneumonia (ICD-10 J15.9), Lobar pneumonia, unspecified organism (ICD-10 J18.1), Bronchopneumonia, unspecified organism (ICD-10 J18.0), and Pneumonia in diseases classified elsewhere (ICD-10 J17).

f Coccidioidomycosis (ICD-10 B38), Histoplasmosis (ICD-10 B39), Blastomycosis (ICD-10 B40), Zygomycosis (ICD-10 B46), other mycoses, not elsewhere classified (ICD-10 B48), Aspergillosis (ICD-10 B44), Invasive pulmonary aspergillosis (ICD-10 B44.0), Unspecified Aspergillosis (ICD-10 B44), Other forms of Aspergillosis (ICD-10 B44.8), Other pulmonary Aspergillosis (ICD-10 B44.1), and disseminated aspergillosis (ICD-10 B44.7).

g Nocardiosis (ICD-10 A43), Nocardiosis, unspecified (ICD-10 A43.9), *Nocardia* sp. identified in Specimen by Organism-specific culture (Code 43365-6), *Nocardia* sp. identified in Isolate (Code 58596-2), Actinomycosis (ICD-10 A42), Other forms of nocardiosis (ICD-10 A43.8), Pulmonary nocardiosis (ICD-10 A43.0), Pulmonary actinomycosis (ICD-10 A42.0), and Pulmonary mycobacterial infection (ICD-10 A31.0).

h Acute bronchiolitis due to respiratory syncytial virus (ICD-10 J21.0), Acute bronchitis (ICD-10 J20), Acute bronchitis due to respiratory syncytial virus (ICD-10 J20.5), and Respiratory syncytial virus pneumonia (ICD-10 J12.1).

i Enterocolitis due to *C difficile* (ICD-10 A04.7), Enterocolitis due to *C difficile*, not specified as recurrent (ICD-10 A04.72), and Enterocolitis due to *C difficile*, recurrent (ICD-10 A04.71).

### Competing-risk analysis and trends of infectious outcomes

CMV was the most significant infectious complication, with the highest 5-year CIF (17.0%) and steepest annual trend (3.4%). Its burden rose sharply within the first 180 days and continued thereafter, underscoring its persistent course (Figure 1C, Tables 1 and 2). Bacterial pneumonia followed as the next most impactful infection (CIF 9.1%, annual trend 1.8%), showing a steady long-term trajectory that contributed substantially to overall morbidity (Figure 1C, Tables 1 and 2). Other infections occurred less frequently but remained clinically important. Mycoses (CIF 6.7%) and Actinomycosis-*Nocardia*/NTM (5.4%) followed a more gradual, later-onset course, reflecting subacute progression (Figure 1C). Respiratory syncytial virus (RSV), although less common (CIF 4.0%, trend 0.8%), demonstrated a consistent upward trajectory, maintaining relevance throughout follow-up (Tables 1 and 2). Bacteremia (CIF 2.9%, annual trend 0.6%) and clostridium difficile infection (CIF 2.8%, annual trend 0.6%) had small but comparable burdens and appeared more episodic (Figure 1C, Tables 1 and 2). Despite their lower incidence, both remain clinically important as potentially modifiable risks. Pairwise analyses confirmed that CMV’s trajectory differed significantly from all other infections (adjusted *p* < 0.001), and bacterial pneumonia also differed from lower-incidence infections (adjusted *p* < 0.001), underscoring their roles as dominant drivers of post-transplant infectious morbidity.

## Discussion

This multicenter, EHR-based study presents a contemporary, competing-risks analysis of major complications following LTx. By 5 years, nearly one-third of recipients developed serious infections occurring earlier and more steeply than noninfectious events, with significant divergence by day 291. These event-specific estimates provide time-based benchmarks that can guide surveillance strategies, prophylaxis, and quality improvement in clinical practice. The temporal patterns carry direct clinical implications. CMV disease showed a sharp rise within the first 6 months, consistent with peak immunosuppression, but incidence continued beyond this period, raising concerns about late-onset CMV and its links to chronic allograft dysfunction and mortality.^4^, ^5^, ^6^ This suggests that fixed-duration prophylaxis may be insufficient, and more individualized, risk-adapted approaches are needed. Bacterial pneumonia increased steadily across the 5 years, reflecting chronic airway vulnerability and highlighting the need for extended preventive strategies, including vaccination and pulmonary hygiene. Less common but consequential infections, such as mycoses and actinomycosis-nocardiosis/NTM presented later, underscoring the importance of ongoing surveillance tools (e.g., fungal biomarkers, sputum cultures) to support earlier detection.^7^, ^8^ RSV demonstrated a steady trajectory, reinforcing opportunities to integrate seasonal vaccination into long–term transplant care. Infections associated with health care exposures, particularly bacteremia and clostridium difficile infection, were less frequent but clinically significant. Their modest incidence belies their preventability, as they are closely linked to modifiable factors such as central line use and antibiotic exposure.^9^ These findings suggest that stewardship and infection-control interventions are key levers for reducing post-transplant morbidity. Noninfectious complications, malignancy, and mortality also carried significant long-term burdens. Malignancies accumulated gradually, reflecting chronic immunosuppression and supporting the implementation of structured cancer screening protocols.^9^ Mortality reached 10.6% at 5 years, consistent with improvements in early survival but persistent vulnerability to late complications. Together, these patterns highlight the need for lifelong, multidisciplinary care of LTx recipients.^10^

A key strength of this study is the use of a large, real-world EHR dataset. While registry data, such as the International Society for Heart and Lung Transplantation database, provide detailed transplant-specific outcomes (e.g., rejection, chronic lung allograft dysfunction), they do not systematically capture infectious complications. TriNetX, in contrast, offers breadth across health systems, enabling the quantification of infection incidence and trajectories that are often underreported in registries. Our findings, therefore, complement and extend registry analyses, which have highlighted chronic rejection as major long-term drivers of morbidity and mortality, by adding a more detailed view of infectious complications.^11^

This study has limitations. TriNetX lacks donor and operative details, and coding variation across sites may affect estimates. In addition, event capture depends on documented clinical encounters within participating health systems, potentially leading to incomplete ascertainment of events occurring outside the network. The ICD-10 code for Chronic Lung Allograft Dysfunction (J4A) became available only in late 2023, necessitating the use of proxy codes earlier in the study, which may have underestimated the incidence. Despite the use of a standardized ICD-9-to-ICD-10 mapping process within TriNetX based on GEMs plus custom algorithms and curation, diagnostic misclassification remains possible. Although this approach enhances consistency across time, administrative codes cannot fully capture clinical nuance, and some degree of variability in diagnostic accuracy should be considered when interpreting our findings. Because the cohort is derived from more than 100 health systems, we could not account for interinstitutional differences in antimicrobial prophylaxis, diagnostic practices, surveillance strategies, and immunosuppression strategies, all of which may influence event detection, reported incidence and generalization. Organism-level attribution was not feasible because ICD-10 codes do not reliably distinguish bacterial species or resistance patterns. Finally, the deidentified design precludes direct linkage with national cohorts. The lower observed mortality compared with registry estimates likely reflects both EHR data capture and analytic approach. Competing-risk methods estimate the probability of death while accounting for other clinically relevant outcomes, which may better reflect real-world post-transplant trajectories but can yield lower cumulative mortality than traditional survival analyses. Mortality should therefore be interpreted within a competing-outcomes framework rather than as a direct survival benchmark.

In conclusion, competing-risks analysis of a large EHR cohort highlights the distinct timing and trajectories of post-transplant complications. These findings support dynamic, individualized surveillance strategies and complement registry data to inform both clinical practice and future trials. From a quality improvement perspective, incorporating CIF-based benchmarks into dashboards or pathway reviews may help centers adjust follow-up intensity over time and better align care practices with evolving risk.

## CRediT authorship contribution statement

**German A. Contreras**: Conceptualization, study design, data curation, analysis, interpretation of results, drafting and revising the manuscript. **George Golovko**: Statistical methodology, data visualization, critical revision of the manuscript for intellectual content, and final approval of the version to be published.

## Declaration of Competing Interest

The authors declare that they have no known competing financial interests or personal relationships that could have appeared to influence the work reported in this paper.

## Data Availability

TriNetX, as the primary data owner, will facilitate the sharing of data. To request access to the data, please contact support@trinetx.com.

## Footnotes

Appendix A Supplementary data associated with this article can be found in the online version at doi:10.1016/j.jhlto.2026.100536.
