# Supplementary material for: Five-year competing-risk analysis of infectious and noninfectious complications after lung transplantation: Real-world evidence from a multicenter EHR-based cohort
Source: JHLT Open. 2026 Mar 10;12:100536. doi: 10.1016/j.jhlto.2026.100536 (PMC13059295; doi:10.1016/j.jhlto.2026.100536)
Supplement: Supplementary file 1 — Supplementary material [file mmc1.docx]

Five-Year Competing Risk Analysis of Infectious and Non-Infectious Complications After Lung Transplantation: Real-World Evidence from a Multicenter EHR-Based Cohort

Supporting Information Table of Contents

1. Table ICD-10 and CTP terms used to build the lung transplant cohort, page 2
2. Table 2 ICD terms, used to build competing risk outcomes, page 5
3. Competing risk methods from TriNext platform, page 7
4. Supplementary Table 1, page 11
5. ICD-9 and ICD-10 mapping strategy, page15

Table Supplemental 1 ICD-10 and CTP terms used to build the lung transplant cohort

| Inclusion codes | CPT:32854 | Lung transplant, double (bilateral sequential or en bloc); with cardiopulmonary bypass |
| --- | --- | --- |
|  | CPT:32853 | Lung transplant, double (bilateral sequential or en bloc); without cardiopulmonary bypass |
|  | CPT:32851 | Lung transplant, single; without cardiopulmonary bypass |
|  | CPT:32852 | Lung transplant, single; with cardiopulmonary bypass |
|  | CPT:1006036 | Lung Transplantation Procedures |
|  | CPT:1006038 | Lung transplant, single |
|  | CPT:1006041 | Lung transplant, double (bilateral sequential or en bloc) |
|  | ICD10CM:Z94.2 | Lung transplant status |
|  | ICD10PCS:0BYC | Respiratory System / Transplantation / Upper Lung Lobe, Right |
|  | ICD10PCS:0BYC0Z0 | Transplantation of Right Upper Lung Lobe, Allogeneic, Open Approach |
|  | ICD10PCS:0BYC0Z1 | Transplantation of Right Upper Lung Lobe, Syngeneic, Open Approach |
|  | ICD10PCS:0BYC0Z2 | Transplantation of Right Upper Lung Lobe, Zooplastic, Open Approach |
|  | ICD10PCS:0BYD | Respiratory System / Transplantation / Middle Lung Lobe, Right |
|  | ICD10PCS:0BYD0Z0 | Transplantation of Right Middle Lung Lobe, Allogeneic, Open Approach |
|  | ICD10PCS:0BYD0Z1 | Transplantation of Right Middle Lung Lobe, Syngeneic, Open Approach |
|  | ICD10PCS:0BYD0Z2 | Transplantation of Right Middle Lung Lobe, Zooplastic, Open Approach |
|  | ICD10PCS:0BYF | Respiratory System / Transplantation / Lower Lung Lobe, Right |
|  | ICD10PCS:0BYF0Z0 | Transplantation of Right Lower Lung Lobe, Allogeneic, Open Approach |
|  | ICD10PCS:0BYF0Z1 | Transplantation of Right Lower Lung Lobe, Syngeneic, Open Approach |
|  | ICD10PCS:0BYF0Z2 | Transplantation of Right Lower Lung Lobe, Zooplastic, Open Approach |
|  | ICD10PCS:0BYG | Respiratory System / Transplantation / Upper Lung Lobe, Left |
|  | ICD10PCS:0BYG0Z0 | Transplantation of Left Upper Lung Lobe, Allogeneic, Open Approach |
|  | ICD10PCS:0BYG0Z1 | Transplantation of Left Upper Lung Lobe, Syngeneic, Open Approach |
|  | ICD10PCS:0BYG0Z2 | Transplantation of Left Upper Lung Lobe, Zooplastic, Open Approach |
|  | ICD10PCS:0BYH | Respiratory System / Transplantation / Lung Lingula |
|  | ICD10PCS:0BYH0Z0 | Transplantation of Lung Lingula, Allogeneic, Open Approach |
|  | ICD10PCS:0BYH0Z1 | Transplantation of Lung Lingula, Syngeneic, Open Approach |
|  | ICD10PCS:0BYH0Z2 | Transplantation of Lung Lingula, Zooplastic, Open Approach |
|  | ICD10PCS:0BYJ | Respiratory System / Transplantation / Lower Lung Lobe, Left |
|  | ICD10PCS:0BYJ0Z0 | Transplantation of Left Lower Lung Lobe, Allogeneic, Open Approach |
|  | ICD10PCS:0BYJ0Z1 | Transplantation of Left Lower Lung Lobe, Syngeneic, Open Approach |
|  | ICD10PCS:0BYJ0Z2 | Transplantation of Left Lower Lung Lobe, Zooplastic, Open Approach |
|  | ICD10PCS:0BYK | Respiratory System / Transplantation / Lung, Right |
|  | ICD10PCS:0BYK0Z0 | Transplantation of Right Lung, Allogeneic, Open Approach |
|  | ICD10PCS:0BYK0Z1 | Transplantation of Right Lung, Syngeneic, Open Approach |
|  | ICD10PCS:0BYK0Z2 | Transplantation of Right Lung, Zooplastic, Open Approach |
|  | ICD10PCS:0BYL | Respiratory System / Transplantation / Lung, Left |
|  | ICD10PCS:0BYL0Z0 | Transplantation of Left Lung, Allogeneic, Open Approach |
|  | ICD10PCS:0BYL0Z1 | Transplantation of Left Lung, Syngeneic, Open Approach |
|  | ICD10PCS:0BYL0Z2 | Transplantation of Left Lung, Zooplastic, Open Approach |
|  | ICD10PCS:0BYM | Respiratory System / Transplantation / Lungs, Bilateral |
|  | ICD10PCS:0BYM0Z0 | Transplantation of Bilateral Lungs, Allogeneic, Open Approach |
|  | ICD10PCS:0BYM0Z1 | Transplantation of Bilateral Lungs, Syngeneic, Open Approach |
|  | ICD10PCS:0BYM0Z2 | Transplantation of Bilateral Lungs, Zooplastic, Open Approach |
| Exclusion codes |  |  |
|  | ICD10PCS:0FY00Z2 | Transplantation of Liver, Zooplastic, Open Approach |
|  | ICD10PCS:0FY00Z1 | Transplantation of Liver, Syngeneic, Open Approach |
|  | ICD10PCS:0FY00Z0 | Transplantation of Liver, Allogeneic, Open Approach |
|  | ICD10PCS:0FY0 | Hepatobiliary System And Pancreas / Transplantation / Liver |
|  | ICD10CM:Z94.4 | Liver transplant status |
|  | CPT:00580 | Anesthesia for heart transplant or heart/lung transplant |
|  | CPT:1006332 | Heart/Lung Transplantation Procedures |
|  | CPT:47135 | Liver allotransplantation, orthotopic, partial or whole, from cadaver or living donor, any age |
|  | CPT:1007811 | Liver Transplantation Procedures |
|  | CPT:00796 | Anesthesia for intraperitoneal procedures in upper abdomen including laparoscopy; liver transplant (recipient) |
|  | ICD10PCS:0TY10Z2 | Transplantation of Left Kidney, Zooplastic, Open Approach |
|  | ICD10PCS:0TY10Z1 | Transplantation of Left Kidney, Syngeneic, Open Approach |
|  | ICD10PCS:0TY10Z0 | Transplantation of Left Kidney, Allogeneic, Open Approach |
|  | ICD10PCS:0TY1 | Urinary System / Transplantation / Kidney, Left |
|  | ICD10PCS:0TY00Z2 | Transplantation of Right Kidney, Zooplastic, Open Approach |
|  | ICD10PCS:0TY00Z1 | Transplantation of Right Kidney, Syngeneic, Open Approach |
|  | ICD10PCS:0TY00Z0 | Transplantation of Right Kidney, Allogeneic, Open Approach |
|  | ICD10PCS:0TY0 | Urinary System / Transplantation / Kidney, Right |
|  | CPT:50365 | Renal allotransplantation, implantation of graft; with recipient nephrectomy |
|  | CPT:50360 | Renal allotransplantation, implantation of graft; without recipient nephrectomy |
|  | ICD10CM:Z94.0 | Kidney transplant status |
|  | CPT:1008109 | Renal allotransplantation, implantation of graft |
|  | CPT:1008098 | Renal Transplantation Procedures |
|  | CPT:00868 | Anesthesia for extraperitoneal procedures in lower abdomen, including urinary tract; renal transplant (recipient) |
|  | CPT:33929 | Removal of a total replacement heart system (artificial heart) for heart transplantation (List separately in addition to code for primary procedure) |
|  | CPT:33935 | Heart-lung transplant with recipient cardiectomy-pneumonectomy |
|  | CPT:33945 | Heart transplant, with or without recipient cardiectomy |
|  | CPT:00580 | Anesthesia for heart transplant or heart/lung transplant |
|  | CPT:1006332 | Heart/Lung Transplantation Procedures |
|  | CPT:33929 | Removal of a total replacement heart system (artificial heart) for heart transplantation (List separately in addition to code for primary procedure) |
|  | CPT:33935 | Heart-lung transplant with recipient cardiectomy-pneumonectomy |
|  | CPT:33945 | Heart transplant, with or without recipient cardiectomy |
|  | ICD10CM:Z94.1 | Heart transplant status |
|  | ICD10CM:Z94.3 | Heart and lungs transplant status |
|  | ICD10PCS:02YA | Heart And Great Vessels / Transplantation / Heart |
|  | ICD10PCS:02YA0Z0 | Transplantation of Heart, Allogeneic, Open Approach |
|  | ICD10PCS:02YA0Z1 | Transplantation of Heart, Syngeneic, Open Approach |
|  | ICD10PCS:02YA0Z2 | Transplantation of Heart, Zooplastic, Open Approach |
|  | ICD10CM:Z94.83 | Pancreas transplant status |
|  | ICD10CM:Z94.82 | Intestine transplant status |
|  | ICD10CM:Z94.9 | Transplanted organ and tissue status, unspecified |
|  | ICD10CM:Z94.8 | Other transplanted organ and tissue status |
|  | ICD10CM:Z94.5 | Skin transplant status |
|  | ICD10CM:Z94.7 | Corneal transplant status |
|  | ICD10CM:Z94.6 | Bone transplant status |
|  | ICD10CM:Z94.81 | Bone marrow transplant status |
|  | ICD10CM:Z94.84 | Stem cells transplant status |
|  | ICD10CM:Z94.89 | Other transplanted organ and tissue status |

Table Supplemental, 2 ICD terms, used to build competing risk outcomes

| Category | Description | ICD-10 Code |
| --- | --- | --- |
| Complications of transplanted organs | Complications of transplanted organs and tissue | T86 |
| Lung transplant rejection | Lung transplant rejection | T86.818 |
| Bronchiolitis | Bronchiolitis obliterans and bronchiolitis obliterans syndrome | J44.81 |
| Pneumothorax and air leak | Pneumothorax and air leak | J93 |
| Other pneumothorax and air leak | Other pneumothorax and air leak | J93.8 |
| Other pneumothorax | Other pneumothorax | J93.83 |
| Pneumothorax, unspecified | Pneumothorax, unspecified | J93.9 |
| Hemothorax | Hemothorax | J94.2 |
| Pyothorax with fistula | Pyothorax with fistula | J86.0 |
| Skin Malignancies | Melanoma and other malignant neoplasms of skin | C43-C44 |
| Lymphoid Malignancies | Malignant neoplasms of lymphoid, hematopoietic, and related tissue | C81-C96 |
| Cytomegaloviral Diseases | Other cytomegaloviral diseases | B25.8 |
| Cytomegaloviral Diseases | Cytomegaloviral disease | B25 |
| Cytomegaloviral Diseases | Cytomegaloviral pneumonitis | B25.0 |
| Cytomegaloviral Diseases | Cytomegaloviral hepatitis | B25.1 |
| Cytomegaloviral Diseases | Cytomegaloviral pancreatitis | B25.2 |
| Cytomegaloviral Diseases | Cytomegaloviral mononucleosis with polyneuropathy | B27.11 |
| Cytomegaloviral Diseases | Cytomegaloviral mononucleosis with meningitis | B27.12 |
| Bacterial Pneumonia | Bacterial pneumonia, not elsewhere classified | J15 |
| Bacterial Pneumonia | Unspecified bacterial pneumonia | J15.9 |
| Bacterial Pneumonia | Lobar pneumonia, unspecified organism | J18.1 |
| Bacterial Pneumonia | Bronchopneumonia, unspecified organism | J18.0 |
| Bacterial Pneumonia | Pneumonia in diseases classified elsewhere | J17 |
| Mycoses | Coccidioidomycosis | B38 |
| Mycoses | Histoplasmosis | B39 |
| Mycoses | Blastomycosis | B40 |
| Mycoses | Zygomycosis | B46 |
| Mycoses | Other mycoses, not elsewhere classified | B48 |
| Mycoses | Aspergillosis | B44 |
| Mycoses | Invasive pulmonary aspergillosis | B44.0 |
| Mycoses | Other pulmonary Aspergillosis | B44.1 |
| Mycoses | Disseminated aspergillosis | B44.7 |
| Mycoses | Other forms of Aspergillosis | B44.8 |
| Clostridium difficile Infection | Enterocolitis due to Clostridium difficile | A04.7 |
| Clostridium difficile Infection | Enterocolitis due to Clostridium difficile, not specified as recurrent | A04.72 |
| Clostridium difficile Infection | Enterocolitis due to Clostridium difficile, recurrent | A04.71 |
| Nocardiosis | Nocardiosis | A43 |
| Nocardiosis | Nocardiosis, unspecified | A43.9 |
| Nocardiosis | Other forms of nocardiosis | A43.8 |
| Nocardiosis | Pulmonary nocardiosis | A43.0 |
| Actinomycosis | Actinomycosis | A42 |
| Actinomycosis | Pulmonary actinomycosis | A42.0 |
| Mycobacterial Infection | Pulmonary mycobacterial infection | A31.0 |

**How do I a run a Competing Risks analysis?**

**https://support.trinetx.com/hc/en-us/articles/360051956673-How-do-I-a-run-a-Competing-Risks-analysis**

Our Competing Risks feature lets you understand a cohort patient's likelihood of experiencing [each one of several mutually exclusive outcomes](https://support.trinetx.com/hc/en-us/articles/360051956673-How-do-I-a-run-a-Competing-Risks-analysis#h_01EQ1QZNQANNFYWX46X3Y5W1ZA). As with Analyze Outcomes, you’ll select a single cohort and an index event. Unlike Analyze Outcomes, you'll select *at least two* outcomes.

The analysis yields two results:

- A plot of Aalen-Johansen cumulative incidence curves
- Outcome Statistics

**The Aalen-Johansen cumulative incidences plot**

The Aalen-Johansen plot illustrates the cumulative incidence of each outcome. Clicking on the graph will enlarge it and show estimated [95% confidence intervals](https://support.trinetx.com/hc/en-us/articles/360051956673-How-do-I-a-run-a-Competing-Risks-analysis#h_01EV9FR5WQCVXJYG5RGBVCD76B) for each point on the curves. Cumulative incidence is defined as the probability that a given outcome has occurred before a given time. It is also sometimes referred to as “incidence proportion,” “risk,” or “attack rate.”

**Outcome Statistics**

Outcome statistics includes the following for each outcome of interest:

- **Outcome name**: The name of the outcome or group of outcomes
- **Patient Count:**The number of patients in the cohort that had this outcome first among all listed outcomes
- **Percentage of Cohort:**The percentage of the cohort represented by the patient count
- **Cumulative Incidence at End of Time Window:**The Aalen-Johansen estimate of the cumulative incidence of this particular outcome at the end of the time window

**What are competing risks?**

"Competing risks" refers to a type of survival analysis concerned with multiple outcomes, where experiencing any *one* terminates a patient's survival time for the purposes of the analysis. An example would be following users of a new medication that potentially increased the risk of both stroke and heart attack, where having either outcome would remove the patient from the cohort. How do we best determine the likelihood of these outcomes independently?

One way many data analysts approach this question is to perform multiple Kaplan-Meier analyses on the same cohort: one analysis for each outcome of interest. One disadvantage of this approach (besides needing multiple analyses) is that treating competing risks as censorings will systematically overestimate the risk of the event of interest. Thus, while this approach might be appropriately conservative in some cases, for a use case like pharmacovigilance it would lead to an overestimation of the risks of adverse events. This is the result of violating the Kaplan-Meier assumption that all censorings are non-informative.

In a traditional survival analysis, the assumption is that there is only one “failure mode” or “death” where patients leave the cohort due to having some outcome. All other ways that a patient could be removed from the cohort are assumed to be “uninformative” or “administrative” – the patient moved away, changed medical providers, or had some unrelated event like a traffic accident that removed them from the study. The benefit of the Aalen-Johansen estimator is that all the estimated cumulative incidences are updated correctly when a patient leaves the cohort due to one of the competing outcomes.

For a single outcome, the Aalen-Johansen estimator reduces to the Kaplan-Meier estimator and would give a cumulative incidence estimate equal to (1 – the Kaplan-Meier survival estimate).

 How are the confidence intervals for Competing Risks calculated?

The Competing Risks analytics feature uses the R Survival library v3.2-3 [1] to calculate the Aalen-Johansen estimate of the cumulative incidence along with 95% confidence intervals.

We give Survival a command of the form:

survfit(Surv(data$time, data$events)~1, weights=data$weights, conf.type=’log-log’, stype=1, ctype=2)

The type parameters indicate that we choose to use a direct Kaplan-Meier calculation of the survival curve (rather than using the Nelson-Aalen cumulative hazard) and the Fleming-

Harrington correction for tied times. We also use the standard log-log transformation of the confidence intervals to avoid collisions with the probability interval endpoints (0 and 1).

Two things are important to note about the Survival package’s standard error calculation. The first is that it treats weights as sampling weights rather than multiplicities (frequency weights). This means that each data entry is treated as a single patient with more or less influence on the cumulative incidence. As a result, the variance will decrease more slowly as the cohort size grows than it would if the weights were treated as multiplicities. The second is that Survival uses a robust estimator of variance called the infinitesimal jackknife (IJ) [2]. It is also called the sandwich estimator, and as we will see, is the same as SAS’ Delta variance estimator derived using the delta method.

In order to validate the calculations, we have compared the Survival output with LIFETEST from SAS v9.4. The relevant parameters are

METHOD=KM CONFTYPE=LOGLOG

FREQ weights ERROR=DELTA or AALEN

We wanted to understand how the two SAS error estimates (DELTA and AALEN) compared to each other and to Survival’s estimate. We also wanted to isolate the potential effects of several features of competing risk data: censoring, different outcomes occurring at the same time (tied times), and multiple events of the same outcome at the same time. Below we show calculations performed on three datasets from TriNetX’s test suite designed to highlight different details.

In Fig. 1 we show the cumulative incidence and confidence intervals for a single outcome of a simple competing risks test dataset. This dataset had no censoring, no tied times, and no event duplication. We see that the cumulative incidence is identical for all three approaches and that the SAS Delta and Survival IJ error estimates are the same.


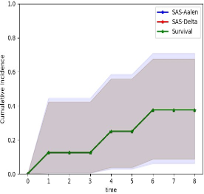


Fig. 1: The cumulative incidence (starred lines) and pointwise confidence intervals (shaded regions) for a single outcome. The stars in the cumulative incidence curves indicate the timepoints at which the cumulative incidence values and confidence intervals are calculated. These test data did not contain censoring, tied times, or event weights > 1.

In Figure 2 we introduce censoring events and tied times across different outcomes to the dataset. Once again, the cumulative incidence values are consistent, and the SAS Delta and Survival IJ errors are the same.


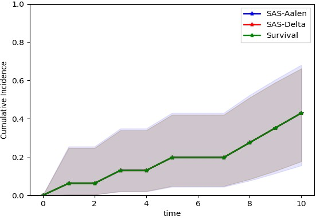


*Fig. 2: The cumulative incidence (starred lines) and pointwise confidence intervals (shaded regions) for a single outcome. The stars in the cumulative incidence curves indicate the timepoints at which the cumulative incidence values and confidence intervals are calculated. These test data do not contain event weights > 1, but they do contain censoring and tied time events.*

In Figure 3, we show the results of a dataset that is the same as the one from Fig. 2 but with duplicate events added (event weights randomly increased from 1). Now we see that while the cumulative incidence estimates remain consistent, the Survival IJ error estimate is noticeably larger than either SAS estimate. This is consistent with Survival treating weights as sampling weights versus SAS treating them as frequencies.


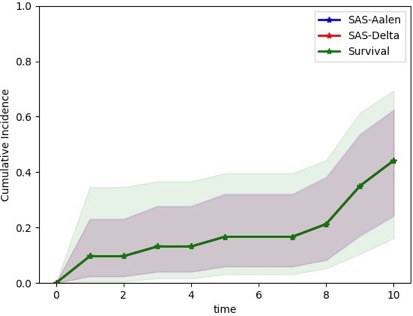


*Fig. 3: The cumulative incidence (starred lines) and pointwise confidence intervals (shaded regions) for a single outcome. The stars in the cumulative incidence curves indicate the timepoints at which the cumulative incidence values and confidence intervals are calculated. These test data are the same as in Fig. 2 but with random integer event weights.*

As we see in the above examples, the cumulative incidence point estimates were robust across SAS and Survival, with or without censoring, tied times, and duplicate events. For the error calculations, we conclude that SAS’ Delta method is clearly equivalent to Survival’s infinitesimal jackknife. However, due to Survival’s treatment of weights as sampling weights, duplicate events cause the Survival confidence intervals to be systematically larger than SAS’.

1. Therneau T (2020). *A Package for Survival Analysis in R*. R package version 3.2- 3, [https://CRAN.R-project.org/package=survival](https://cran.r-project.org/package%3Dsurvival).
2. Terry M. Therneau, Patricia M. Grambsch (2000). *Modeling Survival Data: Extending the Cox Model*. Springer, New York. ISBN 0-387-98784

Supplementary Table 1. Baseline Characteristics of Lung Transplant Recipients

| **Variable** | **Patients n=10,648** | **%** |
| --- | --- | --- |
| Demographics |  |  |
| Age at Index (years) | 58.7 ± 14 | 100% |
| Male | 5788 | 54% |
| Female | 4627 | 43% |
| Unknown Gender | 233 | 2% |
| Not Hispanic or Latino | 8074 | 76% |
| Unknown Ethnicity | 1798 | 17% |
| Hispanic or Latino | 776 | 7% |
| Black or African American | 1253 | 12% |
| White | 7374 | 69% |
| Unknown Race | 1356 | 13% |
| Other Race | 341 | 3% |
| Comorbidities |  |  |
| Hypertensive diseases | 3132 | 29% |
| Ischemic heart diseases | 1943 | 18% |
| Heart failure | 1085 | 10% |
| Endocarditis | 43 | 0% |
| Cerebrovascular diseases | 918 | 9% |
| Peripheral vascular diseases | 429 | 4% |
| Lung diseases due to external agents | 441 | 4% |
| Cystic fibrosis | 625 | 6% |
| Chronic obstructive pulmonary disease | 2234 | 21% |
| Pulmonary heart disease and diseases of pulmonary circulation | 2026 | 19% |
| Interstitial lung disease | 2225 | 21% |
| Diabetes mellitus | 1384 | 17% |
| Body weight | 178 ± 44.1 | 64% |
| Emphysema | 1384 | 13% |
| Bronchiectasis | 1130 | 11% |
| Chronic kidney disease (CKD) | 1053 | 10% |
| Diseases of liver | 794 | 7% |
| Peptic ulcer disease | 44 | 0% |
| Systemic connective tissue disorders | 676 | 6% |
| Malignant neoplasms of lymphoid, hematopoietic, and related tissue | 170 | 2% |
| Solid Neoplasms | 1664 | 16% |
| Dementia | 31 | 0% |
| Mental, Behavioral, and Neurodevelopmental disorders | 2583 | 24% |
| History of infectious diseases in the last year |  |  |
| Sepsis | 52 | 0% |
| Cytomegaloviral disease | 361 | 3% |
| Tuberculosis | 29 | 0% |
| Influenza and pneumonia | 1399 | 13% |
| Human immunodeficiency virus infection | 0 | 0% |
| Candidiasis | 325 | 3% |
| Aspergillosis | 317 | 3% |
| Histoplasmosis | 10 | 0% |
| Coccidioidomycosis | 11 | 0% |
| Cryptococcosis | 10 | 0% |
| Zygomycosis | 13 | 0% |
| Blastomycosis | 10 | 0% |
| History of resistance to antimicrobial drugs infections | 220 | 2% |
| Medications |  |  |
| Immune suppressants† | 2401 | 23% |
| Tacrolimus | 1431 | 13% |
| Mycophenolate mofetil | 1355 | 13% |
| Mycophenolic acid | 525 | 5% |
| Belatacept | 10 | 0% |
| Azathioprine | 375 | 4% |
| Basiliximab | 208 | 2% |
| Cyclosporine | 134 | 1% |
| Sirolimus | 153 | 1% |
| Glucocorticoids | 4491 | 42% |
| Rituximab | 127 | 1% |
| Antineoplastics | 486 | 5% |
| Antimicrobials |  |  |
| Penicillins and beta-lactam antimicrobials | 2580 | 24% |
| Vancomycin | 1055 | 10% |
| Trimethoprim | 1830 | 17% |
| Metronidazole | 296 | 3% |
| Quinolones | 1419 | 13% |
| Erythromycins/macrolides | 2199 | 21% |
| Sulfonamide/related antimicrobials | 1780 | 17% |
| Tetracyclines | 722 | 7% |
| Aminoglycosides | 585 | 5% |
| Lincomycins | 210 | 2% |
| Antivirals |  |  |
| Acyclovir | 299 | 3% |
| Valacyclovir | 265 | 2% |
| Valganciclovir | 519 | 5% |
| Ganciclovir | 82 | 1% |
| Antifungals | 1513 | 14% |
| Fluconazole | 377 | 4% |
| Micafungin | 135 | 1% |
| Voriconazole | 329 | 3% |
| Amphotericin b | 146 | 1% |
| Posaconazole | 167 | 2% |
| Isavuconazonium | 66 | 1% |
| Caspofungin | 53 | 0% |
| Itraconazole | 159 | 1% |
| Anidulafungin | 10 | 0% |
| Flucytosine | 10 | 0% |
| Persons encountering health services for examinations | 3775 | 35% |
| Vaccines | 3260 | 31% |
| Surgery | 5625 | 65% |
| Surgical Procedures on the Cardiovascular System | 3953 | 37% |
| Surgical Procedures on the Digestive System | 794 | 7% |
| Surgical Procedures on the Respiratory System | 1063 | 10% |
| Surgical Procedures on the Integumentary System | 384 | 4% |
| Surgical Procedures on the Musculoskeletal System | 213 | 2% |
| Surgical Procedures on the Nervous System | 170 | 2% |
| Surgical Procedures on the Female Genital System | 52 | 0% |

Note: SD = standard deviation. Percentages are based on the total patient population unless otherwise specified. Continuous variables are presented as means ± standard deviations; categorical variables are represented as counts (percentages). Percentages reflect records with available data. † Immunosuppressed medications used 365 days to 1 day before the index event.

**How are ICD-9 terms mapped to ICD-10 terms?**

The International Statistical Classification of Diseases and Related Health Problems (ICD) is one of the most widely used classification systems for coding diagnosis data. ICD-10 (tenth revision) is currently the international standard and used in more than a hundred countries. ICD-9, the previous revision, is still used in some countries. ICD-11, the most recent revision, came into effect January 1, 2022, but the United States will not be among the early adopters of the new revision. Many countries have created extensions to ICD-10, such as ICD-10 Clinical Modification (ICD-10-CM) in the United States. ICD-10-CM is the classification system used by TriNetX to represent diagnoses. ICD-9 and other extensions/modifications of ICD are mapped to ICD-10-CM.


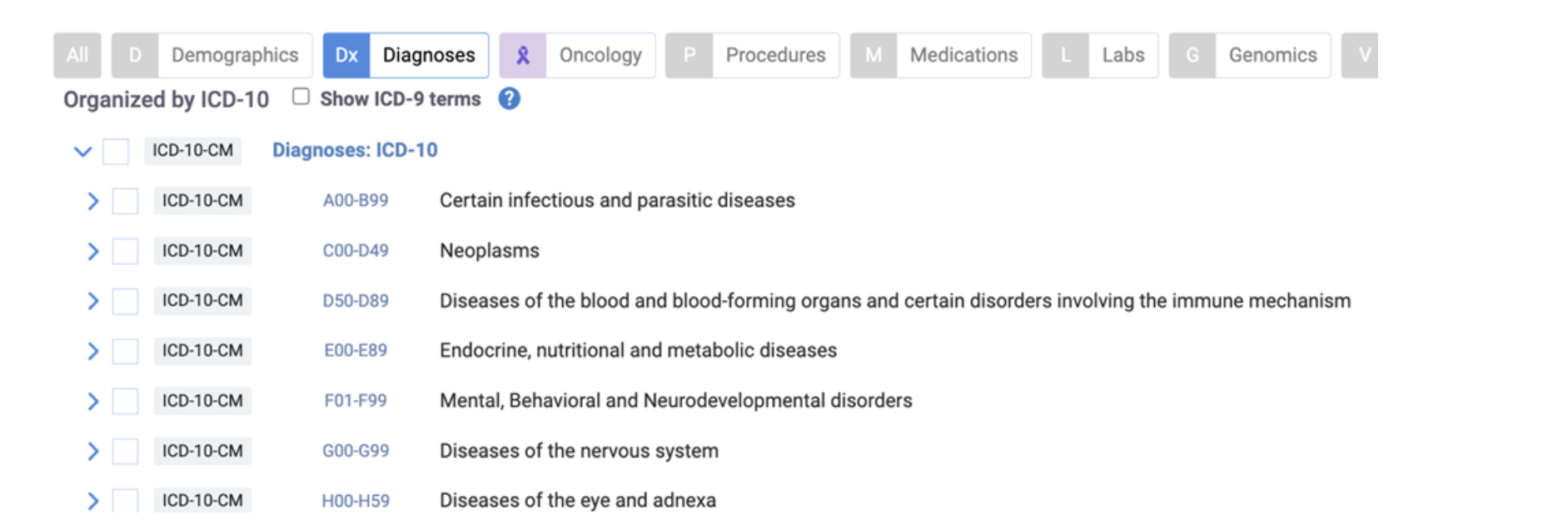


While the default search results for diagnoses are ICD-10-CM terms, users can view ICD-9 to ICD-10 mappings in the hierarchy tree by checking the box to “Show ICD-9 terms.” ICD-9 to ICD-10 mapping is performed by General Equivalence Mappings (GEMs), plus custom algorithms and curation. For example, the ICD-9-CM term 001 “Cholera” is mapped to the ICD-10-CM code for Cholera, A00. ICD-9-CM terms can be distinguished from ICD-10-CM terms because the codes are numeric only whereas ICD-10-CM codes are alphanumeric.


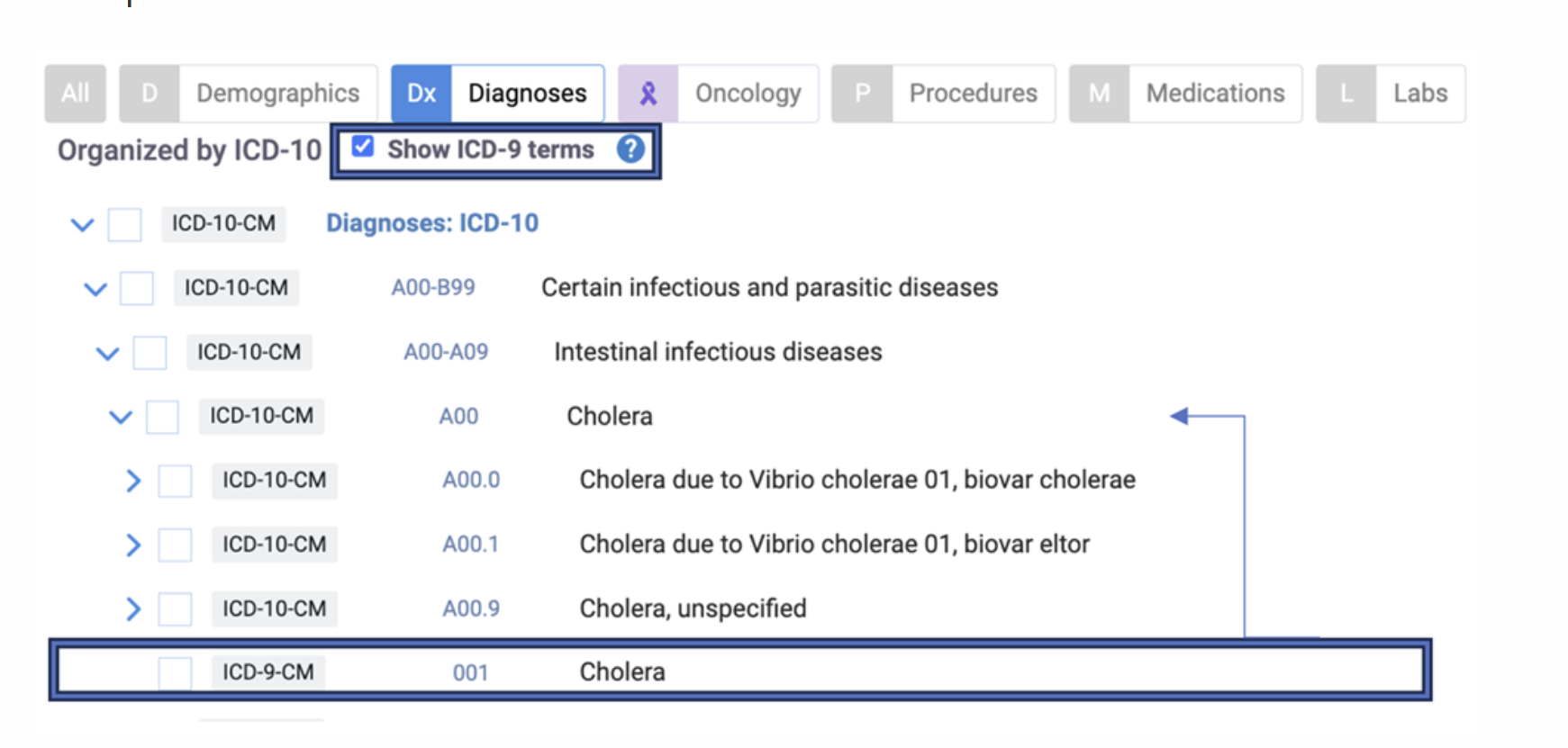


After selecting “Show ICD-9 terms,” users can interact with the ICD-9 terms on their own (e.g., if a user wants to exclude a particular ICD-9 term). This can be helpful when working with some rare diseases where the ICD-9 term is broader than its corresponding ICD-10 term. The section “Correcting Inflated Counts Due to ICD-9 Mapping” in the Best Practices in Querying Rare Diseases Strategy Guide provides more information. Additionally, the Query Builder 101 Training Guide can be a useful resource for understanding how best to use the Query Builder, including ICD-9 to ICD-10 mapping.
